# Ion Transport and Process of Water Dissociation in Electromembrane System with Bipolar Membrane: Modelling of Symmetrical Case

**DOI:** 10.3390/membranes13010047

**Published:** 2022-12-29

**Authors:** Stanislav Melnikov

**Affiliations:** Physical Chemistry Department, Kuban State University, 350040 Krasnodar, Russia; melnikov.stanislav@gmail.com

**Keywords:** bipolar membrane, mathematical modelling, water dissociation, water-splitting kinetics

## Abstract

A model is proposed that describes the transfer of ions and the process of water dissociation in a system with a bipolar membrane and adjacent diffusion layers. The model considers the transfer of four types of ions: the cation and anion of salt and the products of water dissociation—hydrogen and hydroxyl ions. To describe the process of water dissociation, a model for accelerating the dissociation reaction with the participation of ionogenic groups of the membrane is adopted. The boundary value problem is solved numerically using COMSOL^®^ Multiphysics 5.5 software. An analysis of the results of a numerical experiment shows that, at least in a symmetric electromembrane system, there is a kinetic limitation of the water dissociation process, apparently associated with the occurrence of water recombination reaction at the of the bipolar region. An interpretation of the entropy factor (*β*) is given as a characteristic length, which shows the possibility of an ion that appeared because of the water dissociation reaction to be removed from the reaction zone without participating in recombination reactions.

## 1. Introduction

Bipolar electrodialysis is an electromembrane process used for converting salts into the corresponding acids and bases [1]. Historically, the first industrial application of bipolar electrodialysis was the regeneration of a mixture of hydrofluoric and nitric acids [2]. Currently, bipolar electrodialysis is much more widely used for the synthesis of weak organic acids [3,4,5], as well as in several other special applications [6,7].

**Figure 1:**
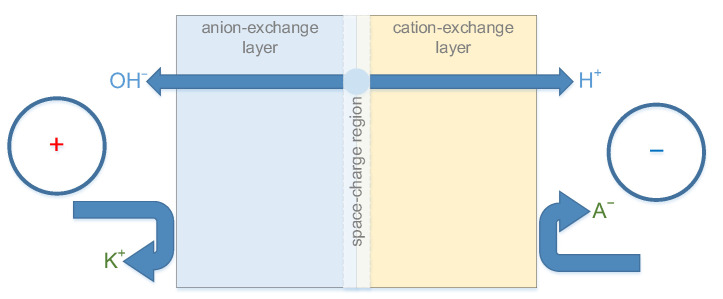
Schematic depiction of a bipolar membrane under reverse bias conditions.

Bipolar electrodialysis implementation requires a special type of ion-exchange membranes—bipolar membranes. A key feature of the bipolar membrane is the ability to accelerate the reaction of water dissociation (also known as the “water-splitting reaction”) with high efficiency. The products of the water dissociation reaction (hydrogen and hydroxyl ions) are the main charge carriers in the bipolar membrane. The transfer of other cations and anions (generally referred to as “salt ions”) should be negligible. These conditions are achieved only with the correct orientation of the bipolar membrane in the electric field (Figure 1). By analogy with semiconductor devices, the orientation that supports the generation of H^+^/OH^−^ ions is called “reverse bias”.

Peculiarities of ions transport in systems with bipolar membranes have been an object of interest for researchers since their emergence to the present day.

The early mathematical models that describe the current–voltage characteristic of a bipolar membrane were based on the assumption of the space–charge region appearance at the cation-exchanger/anion-exchanger interface, analogues with the *p*-*n* junction region in semiconductors (depleted layer model) [8,9,10,11,12,13,14]. According to these type of models, the concentration of mobile ions in the space charge region is very low compared with the concentration of fixed ions. The width of the space–charge region is several nanometers. Considering that a potential drop across the region varies from tenths of a volt (in equilibrium conditions) to several volts (under polarization by electric field), the resulting electric field strength reaches up to (10–100) × 10^6^ V/m.

There are various models for the structure of the cation-exchanger/anion-exchanger contact region (also called “bipolar boundary”). One of the most common models is the abrupt junction model [15,16]. In addition, the bipolar boundary can be represented as a transition zone, in which the concentration of fixed groups smoothly changes from one layer to another [17]. The third option is the “neutral layer” models, which assume that a certain region between the monopolar layers do not contain fixed groups [18,19]. An extension of the neutral layer model is a model in which a catalyst for the water dissociation reaction is placed between monopolar layers; a catalyst can be a dielectric [20,21], ionic [22,23,24], or an electronic conductor [25,26,27].

The overall rate of water dissociation reaction observed in an electromembrane system with a bipolar membrane can be up to 10^7^ times higher than in an aqueous solution. The increase of the water dissociation overall rate is related to the increase in the rate constant of the water dissociation reaction in high strength electric fields that exist in the space charge region. At least three approaches that explain this increase can be found in the literature.

First, the electric field of a functional group weakens the H–OH bond in a water molecule that is near it. Additional polarization occurs in an external electric field [28]. To take these effects into consideration, various researchers had proposed an exponential dependence of the dissociation reaction (forward) rate constant on the electric field strength [15,16,28,29,30,31]:

(1) $$k_{d}=k_{d}^{0}\exp\left(\beta E\right)$$

where $k_{d}^{0}$ and *k_d_* are the forward reaction rate constants without influence of an electric field and under polarization, exp(*βE*) is an exponential function, and *E* is the electric field strength across space–charge region.

Second, the transfer of hydrogen ions from an ionogenic group under the action of an electric field can occur along a chain of favorably oriented water molecules (cooperative proton transfer). The theoretical confirmation of this idea was given by Mafe et al. [32]. Favorable orientation of molecules is facilitated by the electric field, and the thermal motion of water molecules prevents the alignment of such chains. According to this model, the direct decrease in the energy barrier for the proton transfer is small and does not affect the transfer rate. The acceleration coefficient of the rate constant of the water dissociation reaction exponentially depends on the electric field strength with good accuracy up to 5 × 10^8^ V/m. The region of electric field strength and exponential nature of the rate constant dependence is consistent with the approach proposed by Timashev et al. [28,33].

Third, sufficient works [11,13,16,34,35,36,37] use the Onsager theory for the second Wien effect [38] to describe the process of water dissociation in bipolar membranes. Most commonly, the value of the recombination reaction (backwards) rate constant is considered independent of the field strength.

The theory based on the pure manifestation of the second Wien effect was well suited for describing the properties of early bipolar membranes [6]. However, it was found that the water dissociation reaction also proceeds at the solution/anion-exchange membrane interface [39] Zabolotskii et al. [40] showed that the reaction rate also depends on the chemical structure of a bipolar membrane. These observations made it possible to assume that, besides the action of an electric field, certain chemical reactions have a significant effect on the process of water dissociation. In addition, there are fundamental differences in the structure of the cation-exchanger/anion-exchanger interface, which impose significant restrictions on the applicability of the second Wien effect [6].

In the framework of another approach, the acceleration of the water dissociation reaction occurs due to a decrease in its energy of activation with the participation of the functional groups of the membrane (in this case, the term “catalysis of the water dissociation reaction” is used) [14,30,31,39,41,42,43]. The assumption that the dissociation of water in bipolar membranes is influenced by the functional groups of monopolar layers was proposed by Greben et al. [44] and Simons [39,41].

To describe the water dissociation reaction in the electrolyte solution/anion-exchange membrane system, Simons proposed a two-stage protonation/deprotonation mechanism (Equations (2)–(5)). Simons showed that the rate constant of the limiting step for the reaction involving tertiary amino groups (reactions (4) and (5)) is five orders of magnitude higher than the dissociation rate constant in the absence of ionogenic groups (Equation (6)).

(2) $$\mathrm{AH}+H_{2}O\overset{k_{1}}{\underset{k_{-1}}{⇄}}A^{-}+H_{3}O^{+}$$

(3) $$A^{-}+H_{2}O\overset{k_{2}}{\underset{k_{-2}}{⇄}}\mathrm{AH}+{\mathrm{OH}}^{-}$$

(4) $$B+H_{2}O\overset{k_{1}}{\underset{k_{-1}}{⇄}}{\mathrm{BH}}^{+}+{\mathrm{OH}}^{-}$$

(5) $${\mathrm{BH}}^{+}+H_{2}O\overset{k_{2}}{\underset{k_{-2}}{⇄}}B+H_{3}O^{+}$$

(6) $${2H}_{2}O\overset{k_{d}}{\underset{k_{r}}{⇄}}H_{3}O^{+}+{\mathrm{OH}}^{-}$$

**Figure 2:**
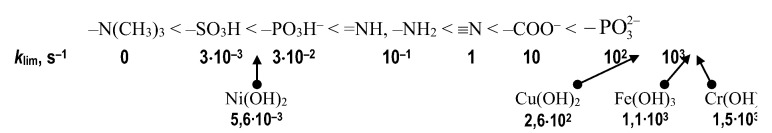
Row of catalytic activity of various functional groups and d-metals hydroxides.

Greben et al. [44] showed that functional groups of different nature (sulfonic, carboxyl, phosphonic) are directly involved in the acceleration of the water dissociation reaction. The studies carried out in [44] showed the dependence of the potential difference across a bipolar membrane on the ionization constant of the functional groups of the cation-exchange layer (Table 1):

Zabolotsky et al. [40,42] calculated the rate constants of limiting stages for various ionogenic groups using data on the equilibrium constants of ionogenic groups (or their analogues in solution) protonation/deprotonation reactions and data on recombination rate constants (*k*_-1_, *k*_-2_). For the mechanisms described by Equations (2)–(5), the limiting stages are the second steps with limiting rate constants *k*_2_. Based on the results obtained in [42], a series of catalytic activity of ionogenic groups in the reaction of water dissociation was built, which qualitatively agrees well with the results of experimental observations. Melnikov et al. [45] expanded this series with some inorganic catalysts, hydroxides of d-elements (Figure 2).

The theoretical description of ion transport in electromembrane systems with bipolar membranes is the subject of a large number of works [8,9,11,13,14,16,19,29,31,32,46,47,48,49,50,51,52,53]. In a number of works, equations have been derived to describe the current–voltage characteristic of a bipolar membrane as a whole [50,54] or only of the space–charge region [30].

Recently, due to the development of computer technology and software products, works have begun to appear that consider the violation of the electrical neutrality condition at the cation-exchanger/anion-exchanger interface [13,16,52]. Instead of the electroneutrality condition, the indicated works used the Poisson equation.

The disadvantage of some of these works is that the reaction of water dissociation occurring at the bipolar boundary is considered as a “fast” reaction. On the one hand, this allows using the quasi-equilibrium condition ($c_{H^{+}}c_{{\mathrm{OH}}^{-}}=10^{-14}$) at any point inside the electromembrane system, which greatly simplifies the solution. At the same time, this formulation of the problem does not consider the chemical overvoltage that occurs in the bipolar membrane because of a slow chemical reaction—the reaction of water dissociation.

The aim of this work is to develop a mathematical model that describes the ion transport and dissociation of water in a symmetrical system with a bipolar membrane using the Nernst–Planck and Poisson equations, considering the catalysis of the water dissociation reaction by ionogenic groups, as well as the recombination reaction of hydrogen and hydroxyl ions.

## 2. Problem Formulation

**Figure 3:**
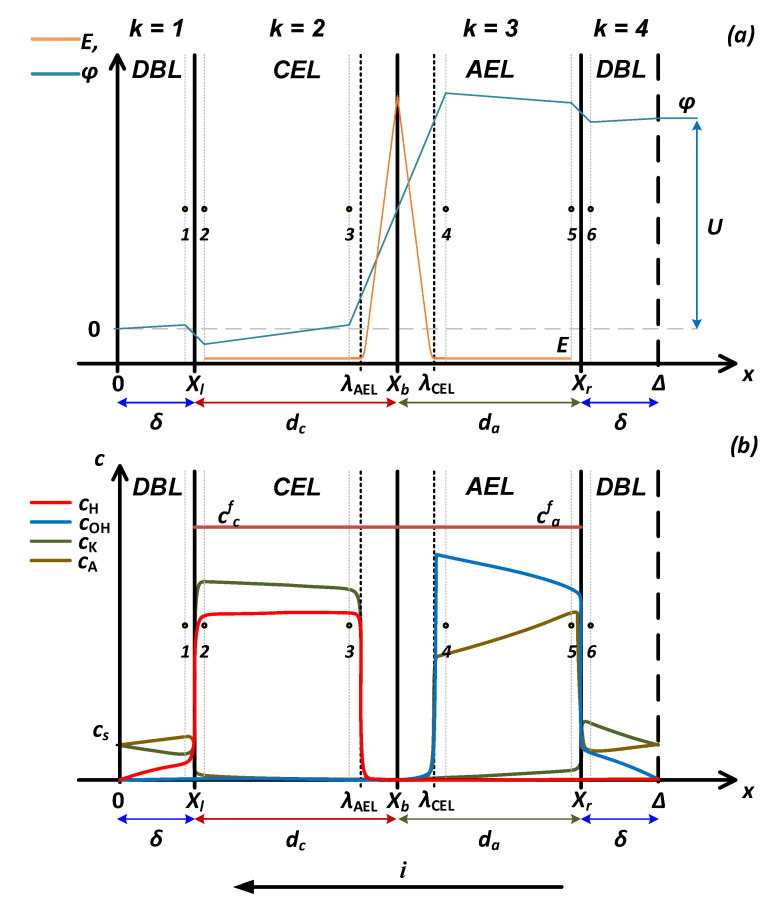
Schematic representation of the potential distribution and electric field strength (**a**) and ion concentration profiles (**b**) in a four-layer electromembrane system. *DBL*—diffusion boundary layer, *CEL*—cation-exchange layer, *AEL*—anion-exchange layer.

The system under study is a bipolar membrane with thicknesses of the anion-exchange and cation-exchange layers being *d_a_* and *d_c_* (layers 2 and 3); diffusion layers with a thickness *δ* are located on both sides of the membrane (layers 1 and 4). The schematic depiction of the system is shown in Figure 3.

There are four types of ions in the system: two cations H^+^ and K^+^ and two anions OH^−^ and Cl^−^. Hereinafter, I will refer to the ions K^+^ and Cl^−^ as “salt ions”.

An electric current flow through the system in such a way that the bipolar membrane is under reverse bias polarization (so-called “generation mode”).

In each of the four layers, the transport of each of the four ions is given by the Nernst–Planck equation (written for the one-dimensional case):

(7) $$j_{i}^{k}=-D_{i}\left(\frac{dc_{i}}{dx}+\frac{z_{i}F}{RT}c_{i}\frac{d\varphi }{dx}\right);\;k=1,2,3,4;\;i=K^{+}{,\;\mathrm{Cl}}^{-}{,\;H}^{+}{,\;\mathrm{OH}}^{-}$$

In what follows, when writing the equations, the following notation is introduced: $C=K^{+},\;A={\mathrm{Cl}}^{-},\;H=H^{+},\;\mathrm{OH}={\mathrm{OH}}^{-}$.

The condition of fluxes stationarity is set for each of the four ions:

(8) $$\frac{d\bar{j_{i}}}{dx}=\frac{dj_{i}}{dx}=\nu_{i}$$

where $\bar{j_{i}}$ are ions fluxes in the membrane (layers 2 and 3); $j_{i}$ are ions fluxes in the solution; $\nu_{i}$ is a net water dissociation reaction rate. For salt ions $\nu_{C}=\nu_{A}=0$, and for hydrogen and hydroxyl ions, $\nu_{H}=\nu_{\mathrm{OH}}=\nu$.

To describe the dependence of the electric potential on the electric charge density, the Poisson equation is used:

(9) $$\varepsilon \varepsilon_{0}\frac{\partial^{2}\varphi }{\partial x^{2}}=-F\left(\sum z_{i}c_{i}\pm c_{f}^{j}\right)$$

Where $c_{f}^{j}$ are the concentrations of fixed ions in the membrane ($c_{f}^{c}$ for the cation-exchange layer and $c_{f}^{a}$ for the anion-exchange layer). For diffusion boundary layers, $c_{f}^{j}=0$.

It is assumed that the condition of chemical equilibrium is satisfied in the depth of the layers:

(10) $$c_{H}c_{\mathrm{OH}}=K_{w}$$

and at the interphase boundaries *X_l_*, *X_r_* and *X_b_*, it is violated due to the localization of the electric field of high intensity.

The system of Equations (7)–(10) is supplemented by the following boundary conditions:

on the left boundary (*x* = 0):

(11) $$c_{i}(0)=c_{i}^{0}$$

(12) φ(0)=0

on the right boundary (*x* = 2*δ* + *d_a_* + *d_c_*):


(13) $$c_{i}(2\delta +d_{c}+d_{a})=c_{i}^{0}$$

(14) $$\varphi (2\delta +d_{c}+d_{a})=U$$

At all interfaces (between points 1 and 2, 3 and 4, 5 and 6 (Figure 3)), the condition of equality of electrochemical potentials is fulfilled:

(15) $$c_{i}(X_{n}-0)e^{\varphi (X_{n}-0)}=c_{i}(X_{n}+0)e^{\varphi (X_{n}+0)}$$

where $\left(X_{n}-0\right)$ are points to the left of the boundary (points 1, 3, 5); $\left(X_{n}+0\right)$ are points to the right of the boundary (points 2, 4, 6); *n* = *l*, *r*, *b* refers to the boundaries cation-exchange layer/solution, anion-exchange layer/solution, and cation-exchange layer/anion-exchange layer.

The current flowing through the system can be expressed in terms of the sum of the ion fluxes:

(16) $$i=F\sum_{i}z_{i}j_{i}$$

The system of Equations (7)–(10) with boundary conditions (11)–(16) is a boundary value problem in a four-layer region.

The problem is a direct boundary value problem, i.e., from the known initial parameters of the model, it is necessary to calculate the current–voltage characteristic of the bilayer membrane.

### 2.1. Kinetics of the Water Dissociation Reaction

Taking into account reactions (4)–(6), the rate of water dissociation with the catalytic participation of ionogenic groups B can be expressed as follows (the equation is given for the rate of generation of a hydrogen ion and an anion-exchange layer):

(17) $$\nu =\nu_{H^{+}}=\nu_{{\mathrm{OH}}^{-}}=k_{2}c_{{\mathrm{BH}}^{+}}c_{H_{2}O}-k_{-2}c_{B}c_{H^{+}}+k_{d}c_{H_{2}O}^{2}-k_{r}c_{H^{+}}c_{{\mathrm{OH}}^{-}}$$

To simplify the calculations, we assume that in the space-charge region almost all ionogenic groups (which participate in the rate-limiting steps of the water dissociation reaction (Equations (3) and (5))) are protonated [55]. The reason for this is a decrease in the concentration of H^+^/OH^−^ ions in the depleted layer, which leads to an increase in the degree of dissociation of ionogenic groups [56].

(18) $$c_{{\mathrm{BH}}^{+}}>>c_{B},\;c_{{\mathrm{OH}}^{-}}>>c_{B}$$

The rate of water autoprotolysis is low compared to the experimentally observed fluxes of H^+^/OH^−^ ions. In this case, we can neglect the second and third terms in Equation (17), thus obtaining:

(19) $$\nu =k_{2}c_{{\mathrm{BH}}^{+}}c_{H_{2}O}-k_{r}c_{H^{+}}c_{{\mathrm{OH}}^{-}}=k_{2}^{*}-k_{r}c_{H^{+}}c_{{\mathrm{OH}}^{-}}$$

where $k_{2}^{*}$ is the effective rate constant of the limiting stage of the catalytic water dissociation reaction. $c_{H_{2}O}$ is large enough to consider it constant, and $c_{{\mathrm{BH}}^{+}}$ can be taken equal to the ion-exchange capacity of the membrane. In the absence of an electric field, the $k_{2}^{*}$ value is constant.

(20) $$k_{2}^{*}=k_{2}c_{{\mathrm{BH}}^{+}}c_{H_{2}O}$$

The second term in Equation (19) is the rate of the water recombination reaction. It depends on the concentration of H^+^/OH^−^ ions, which increase with an increase in the rate of the water dissociation reaction.

Let us assume that $k_{2}^{*}$ exponentially depends on the electric field strength [29,30]:

(21) $$k_{2}^{*}(E)=k_{2}^{*}\exp(\beta E)$$

Finally, for the anion-exchange layer, we obtain:

(22) $$\nu =\nu_{H^{+}}=\nu_{{\mathrm{OH}}^{-}}=k_{2}^{*}(E)-k_{r}c_{H^{+}}c_{{\mathrm{OH}}^{-}}$$

Similar reasoning can be used to write an expression describing the reaction rate of water dissociation in the cation-exchange layer of acid groups AH:

(23) $$\nu =\nu_{{\mathrm{OH}}^{-}}=\nu_{H^{+}}=k_{2}^{*}(E)-k_{r}c_{H^{+}}c_{{\mathrm{OH}}^{-}}$$

where

(24) $$k_{2}^{*}(E)=k_{2}(E)c_{A^{-}}c_{H_{2}O}$$

Expressions (22) and (23) represent the dependence of the water dissociation reaction rate on the electric field strength. Each of them can be used separately in Equation (8) to describe the process of water dissociation in an electromembrane system with a bipolar membrane.

### 2.2. Symmetrical Case and Model Parameters

Within the framework of this article, a symmetric case will be considered, which imposes a number of restrictions on the system under study. The membrane is bathed by a symmetrical electrolyte (KCl) solution with a known concentration of salt ions ($c_{K}=c_{A}=c_{s}$). A concentration of protons and hydroxyl ions in the solution is also known and is equal to $c_{H}=c_{\mathrm{OH}}=c_{W}=\sqrt{K_{w}}$. We will assume that two identical diffusion boundary layers are adjacent to the membrane; we will consider the thicknesses of the cation-exchange and the anion-exchange layers of the membrane to be the same. We will assume that the concentrations of fixed ions in both layers of the bipolar membrane are the same in magnitude, but differ in the sign of the charge. The model does not distinguish any special area in which the catalyst for the water dissociation reaction is located, but it is assumed that the catalytically active functional groups are evenly distributed over the cation-exchange and anion-exchange layers. We will assume that functional groups in both layers have the same activity in the water dissociation reaction. The rate of the water dissociation reaction is expressed by two parameters: the rate constant $k_{2}$ and the entropy factor β.

The main set of parameters that was used in calculations are presented in Table 2.

### 2.3. Numerical Calculations

The COMSOL^®^ Multiphysics 5.5 software package was used to build a one-dimensional physical model and modelling simulations in the framework of Nernst–Planck–Poisson equations. The Tertiary Current Distribution, Nernst–Planck Interface with Poisson charge conservation model, and four dependent variables (concentrations of ionic species) were used.

Ion-exchange layers within the framework of this model are presented as a continuous medium, which is filled with a “virtual electrolyte solution” of mobile and fixed ions. The diffusion coefficients *D_i_* in each layer are specified; the potential drop *U* and the ion concentration *c_i_*^0^ are fixed at the system boundaries.

The standard COMSOL^®^ node Reaction was used to set the net reaction rate of water dissociation according to the Equations (22) and (23).

The MUMPS linear solver and highly nonlinear (Newton) solver were used for calculations.

The simulation solves for the stationary current and concentration distributions for a given potential over the cell.

## 3. Experimental

### 3.1. Bipolar Membranes

To test the adequacy of the model, the calculation results were compared with the current–voltage characteristics of heterogeneous bipolar membranes MB-1, MB-2, MB-3 (JSC Shchekinoazot, Shchekino, Russia). Heterogeneous bipolar membranes were chosen as objects of study, since they consist of monopolar layers of approximately equal thickness and do not contain separately introduced catalytic additives. The difference between these bipolar membranes is due to the different chemical composition of the ion-exchange matrices of the ion exchanger used in their manufacture.

Physicochemical properties of bipolar membranes are presented in Table 3.

For the manufacture of these bipolar membranes, the hot-pressing method of thermoplastic cation- and anion-exchange membranes is used. In the first step the monopolar membranes sheets are made from fine powder composition (consisting of a resin and the inert binder). Then sheets of cation- and anion-exchange membranes are superimposed and subjected to hot press to obtain a laminated membrane sheet. Preparation of heterogeneous BPM by this method results in a heterogeneous bipolar border, where only part of the surface is occupied by the contacts of cation and anion-exchange particles.

### 3.2. Current–Voltage Characteristics Measurement

Since one of the parameters of the model is the thickness of the diffusion layers near the bipolar membrane, the rotating membrane disk method was chosen to study the current–voltage characteristics, which makes it possible to set the thickness of the diffusion layer. This method is described in detail in [59,60].

**Figure 4:**
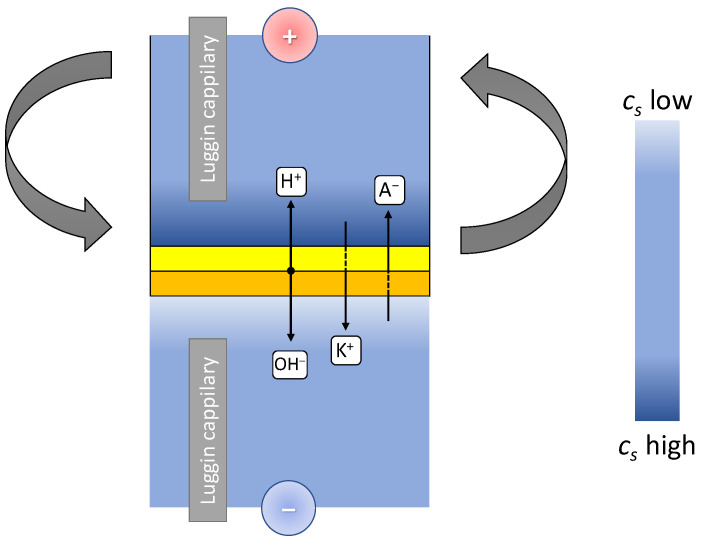
Scheme of ion fluxes through the membrane and distribution of electrolyte concentration and density in a device with a rotating membrane disk.

Figure 4 shows a schematic representation of ion fluxes and the distribution of ion concentrations in the electromembrane system under study.

The rotating membrane disk allows adjustment of the thickness of the diffusion boundary layer near the membrane surface by changing the disk rotation speed. Under such conditions, the membrane surface is equally accessible, and the horizontal orientation of the membrane makes it possible to exclude the influence of thermal and gravitational convection.

The relationship between the thickness of the diffusion layer *δ* and the angular velocity of rotation of the membrane disk *ω* for rotating disk was derived by Levich [61]:

(25) $$\delta =1.61D_{s}^{1/3}\nu^{1/6}\omega^{-1/2}$$

where: *D* is the electrolyte diffusion coefficient; *v* is the kinematic viscosity of the solution; ω is the angular velocity, rad/s.

The experimental data were obtained at disk rotation speed of 100 rpm, which, for aqueous solution, results in diffusion boundary layer thickness of 53 μm. 

## 4. Results and Discussion

**Figure 5:**
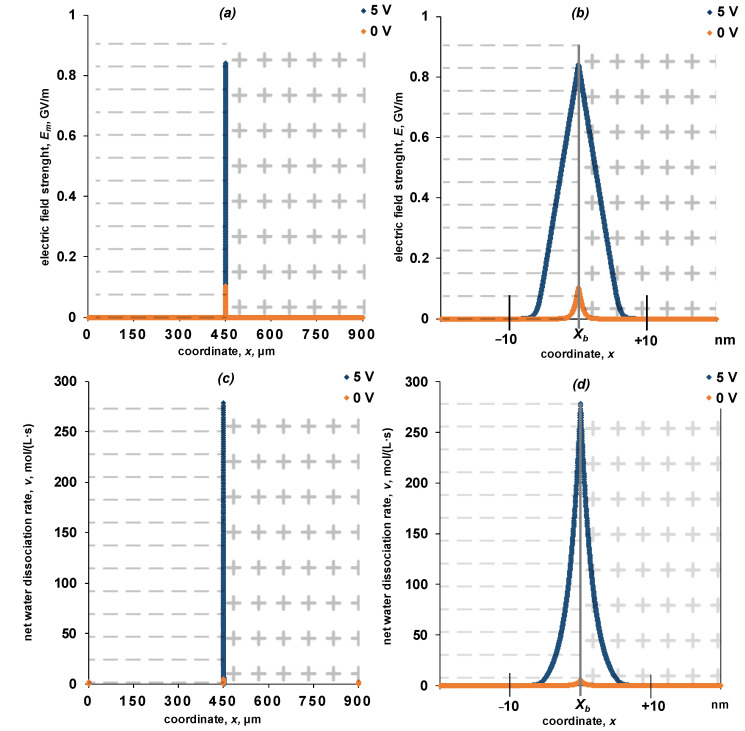
Dependence of the electric field strength (**a**,**b**) and the rate of the water dissociation reaction (**c**,**d**) on the spatial coordinate inside the bipolar membrane (**a**,**c**) and near the point $x=X_{b}$ (**b**,**d**). The calculation is shown for potential drops of 0 and 5 V. The values of the parameters given in Table 2 are used for the calculation. *k*_2_* = 10 M/s, *β* = 4 m/GV.

**Figure 6:**
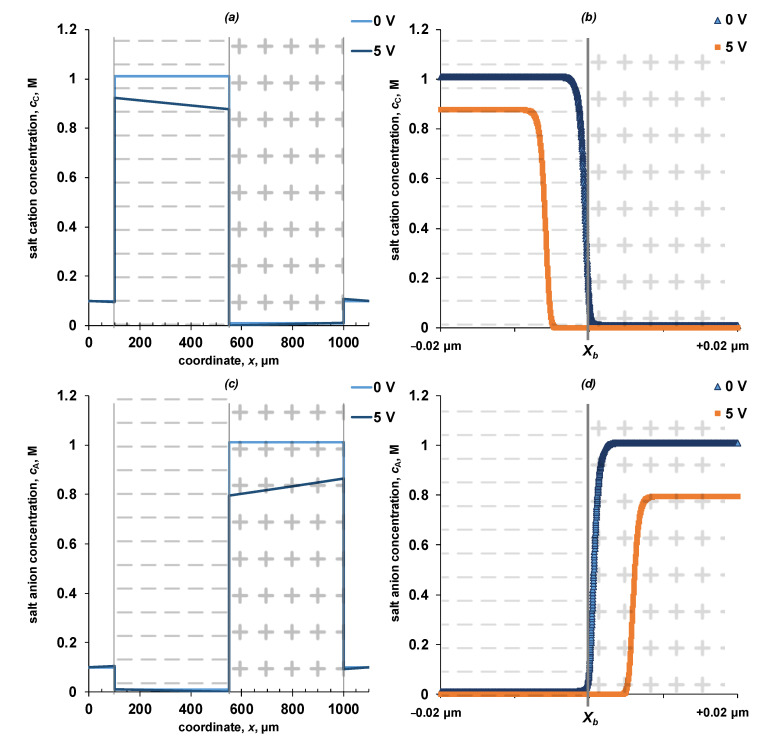
Concentration profiles of salt cations (**a**,**b**) and salt anions (**c**,**d**) in a four-layer electromembrane system (**a**,**c**) and near the point $x=X_{b}$ (**b**,**d**). The calculation is shown for potential drops of 0 and 5 V. The values of the parameters given in Table 2 are used for the calculation. $k_{2}^{*}$ = 10 M/s, *β* = 4 m/GV.

**Figure 7:**
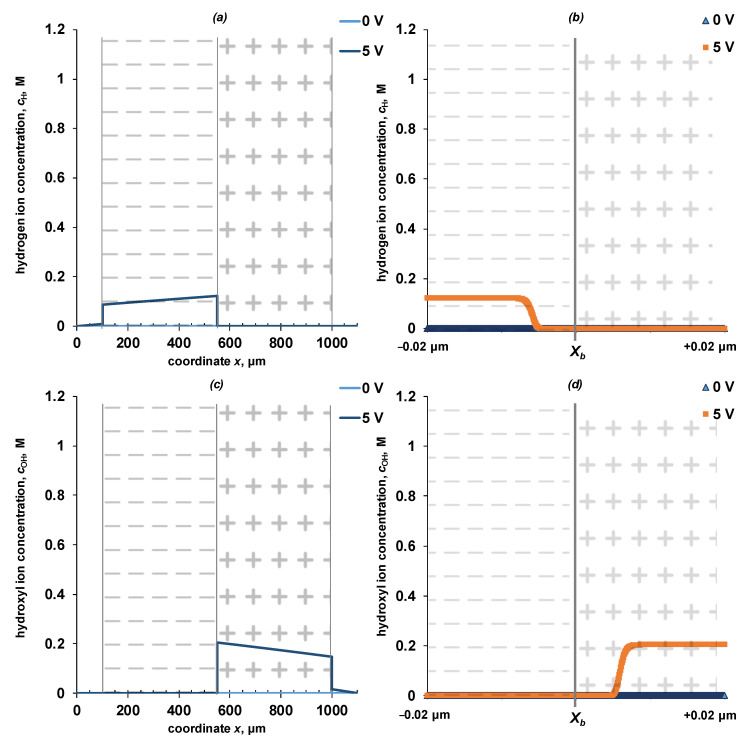
Concentration profiles of hydrogen ions (**a**,**b**) and hydroxyl ions (**c**,**d**) in a four-layer electromembrane system (**a**,**c**) and near the point $x=X_{b}$ (**b**,**d**). The calculation is shown for potential drops of 0 and 5 V. The values of the parameters given in Table 2 are used for the calculation. $k_{2}^{*}$ = 10 M/s, *β* = 4 m/GV.

The distribution of the electric field strength and the water dissociation reaction net rate (Figure 5), and concentration profiles of ions (Figure 6 and Figure 7) on the spatial coordinate for the entire system and near the interface (*x* = *X_b_*) are shown in Figure 5, Figure 6 and Figure 7.

**Figure 8:**
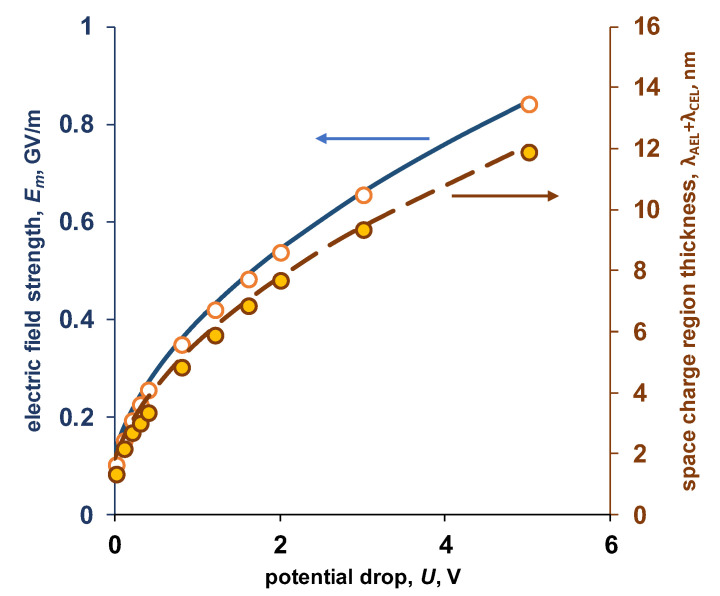
Dependence of the electric field strength at the point *x* = *X_b_* and the width of the space charge region on the potential drop on the electromembrane system under study. Points—numerical calculation, lines—calculation by Equations (26) and (27).

The width of the region in which the space charge is localized in the absence of external polarization is about 2 nm (Figure 5b), which agrees well with the known data [16]. As the potential drop increases, the width of the space charge region increases. For a symmetric system, the width of the space charge region (Equation (26)) and the electric field strength (Equation (27)) obtained from numerical solution coincides with the calculation using the Schottky equation (Figure 8).

(26) $$\lambda_{\mathrm{CEL}}+\lambda_{\mathrm{AEL}}=\sqrt{\frac{2\varepsilon_{r}\varepsilon_{0}}{F}\frac{\left(c_{f}^{c}+c_{f}^{a}\right)}{c_{f}^{c}c_{f}^{a}}\Delta \varphi^{scr}}$$

(27) $$E_{m}=\sqrt{\frac{2F}{\varepsilon_{r}\varepsilon_{0}}\frac{c_{f}^{c}c_{f}^{a}}{\left(c_{f}^{c}+c_{f}^{a}\right)}\Delta \varphi^{scr}}$$

where $\Delta \varphi^{scr}$ is the potential drop across space charge region, located from $\lambda_{\mathrm{AEL}}$ to $\lambda_{\mathrm{CEL}}$ (Figure 3).

Comparison of the electric field strength and the rate of the water dissociation reaction distributions along the spatial coordinate (Figure 5b,d) allows the conclusion that with a noticeable rate, this reaction proceeds only inside the space–charge region near the cation-exchanger/anion-exchanger interface. The reaction rate increases with an increase in the potential drop.

An increase in the electric field strength at the cation-exchanger/anion-exchanger interface is associated with the formation of the so-called “depleted layer”—a region of space in which the concentrations of mobile ions is small (Figure 6 and Figure 7). When polarized by a reverse bias electric current, salt ions are removed from the interface, which leads to a significant increase of the electrical resistance and a sharp increase of the potential drop across the interface. Due to an increase in the electric field strength, a water dissociation reaction zone is formed inside the bipolar region, which can propagate both into the cation-exchange and anion-exchange layers in the studied symmetric system. Due to the water dissociation reaction, new charge carriers H^+^/OH^−^ ions appear, which, respectively, are transferred by the electric field to the cation-exchange and anion-exchange layers. Inside the monopolar layers in the state of equilibrium, the concentration of hydroxyl ions in the anion-exchange layer and hydrogen ions in the cation-exchange layer is five orders of magnitude higher than when the membrane is polarized. The decrease in the concentration of water dissociation products, as co-ions in the corresponding layers of the bipolar membrane, is caused by a partial loss of ions because of the recombination reaction, as well as an increase in the concentration of counterions—water dissociation products inside the layers.

**Figure 9:**
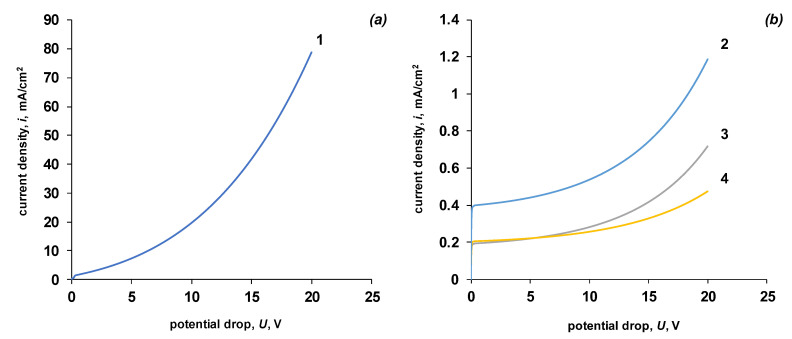
Calculated total current–voltage characteristic (**a**) and partial currents by salt ions (**b**). 1—general current–voltage characteristic, 2—total salt ion current, 3—salt cation current, 4—salt anion current. The values of the parameters given in Table 2 are used for the calculation. $k_{2}^{*}$ = 10 M/s, *β* = 4 m/GV.

The calculated overall current–voltage characteristic of a symmetrical bipolar membrane is shown in Figure 9.

The initial linear region corresponds to the transfer of salt ions from the bipolar region to the external solution. The ion flux in each of the layers in the absence of convective transfer can be expressed as the sum of the diffusion and migration fluxes:

(28) $$J_{j}=-D_{j}z_{j}\frac{dc_{j}}{dx}+t_{j}i$$

At *U* = 0 V, there is no migration transfer of ions in the system and the diffusion fluxes of ions in each of the layers are directed from the cation-exchanger/anion-exchanger interface to the membrane-solution boundary, that is, the diffusion fluxes in different layers of the bipolar membrane are directed in opposite directions and partially mutually exclude each other. In this case, the Donnan potential jump at the bipolar boundary is completely compensated by the sum of two Donnan potentials at the outer boundaries of the solution/cation-exchange layer and the anion-exchange layer/solution [16].

In case a small potential drop is applied to the system (in the case under consideration, 0 < *U* < 0.14 V), the concentration profiles of ions are rearranged in such a way that the migration and diffusion fluxes of salt anions inside the cation-exchange layer become directed in the same direction. In the anion-exchange layer, in which the salt anions are counterions, the migration and diffusion flows are directed in opposite directions. The coincidence of the directions of the migration and diffusion flows in the cation-exchange layer leads to a decrease in the concentration of anions at the bipolar boundary cation-exchanger/anion-exchanger. The same processes appear simultaneously concerning salt cations in the anion-exchange layer. At a certain critical value of the potential jump, the co-ions concentration at the interface becomes low and the current reaches its limiting value. In this case, the formation of the limiting state in the electromembrane system occurs not according to the external diffusion mechanism, as for a monopolar membrane, but according to the innerdiffusion mechanism [9,62]. The “zeroing” of the concentration of salt co-ions to the left and right of the cation-exchanger/anion-exchanger interface also leads to a decrease of the concentration of counterions in the corresponding layers (anions in the anion-exchange layer and cations in the cation-exchange layer). As a result, a space charge region is formed in which ionogenic groups are located without mobile ions compensating their charge. When the electric field strength across the space charge region becomes high enough, the rate of the water dissociation reaction (the first term in Equation (22)) becomes greater than the recombination rate (the second term) and the water dissociation net rate becomes positive. As a result, an increase in current above the limit value is recorded on the current–voltage characteristic.

The magnitude of the electric field strength necessary to achieve a positive value of the net rate of the water dissociation reaction can be estimated using the Equation (22):

(29) $$E^{crit}>\frac{\ln\left(\frac{k_{r}c_{H^{+}}c_{{\mathrm{OH}}^{-}}}{k_{2}c_{{\mathrm{BH}}^{+}}c_{H_{2}O}}\right)}{\beta }$$

Assuming that the value $c_{H^{+}}c_{{\mathrm{OH}}^{-}}$ before the onset of water dissociation differs slightly from the equilibrium value (10^−14^), and using the effective value of the forward reaction rate constant (Equation (21)) we can obtain an approximate relationship:

(30) $$E^{crit}>\frac{-4.343-\ln\left(k_{2}^{*}\right)}{\beta }$$

From the analysis of the expression obtained, it follows that the electric field strength at which the rate of the water dissociation reaction becomes higher than the rate of the reverse reaction depends on the properties of the catalyst—it increases with an increase in the rate constant $k_{2}^{*}$ and decreases with an increase in β.

At very high fluxes of hydrogen and hydroxyl ions (high values of the potential drop across the membrane), an increase in the flux of salt ions also occurs (Figure 9b). The reason for the increase in the flux of salt ions is the increase in the concentration of water dissociation products at the membrane/solution interfaces: the cation exchanger/solution interface is enriched with hydrogen cations, and the anion exchanger/solution interface is enriched with hydroxyl anions. The appearance of ions of the same charge at the membrane/solution interface accelerates the delivery of oppositely charged salt ions to the membrane/solution interface (chloride ions to the cation-exchange layer and sodium ions to the anion-exchange layer). An increase in the concentration of salt ions at the membrane/solution interface leads to an increase in the concentration of co-ions in the corresponding monopolar layers of the bilayer membrane, which in turn increases the total flux of salt ions through the membrane. At extremely high potential jumps (*U* > 10 V), the phenomenon of flux inversion is possible when salt ions again become the main charge carriers.

**Figure 10:**
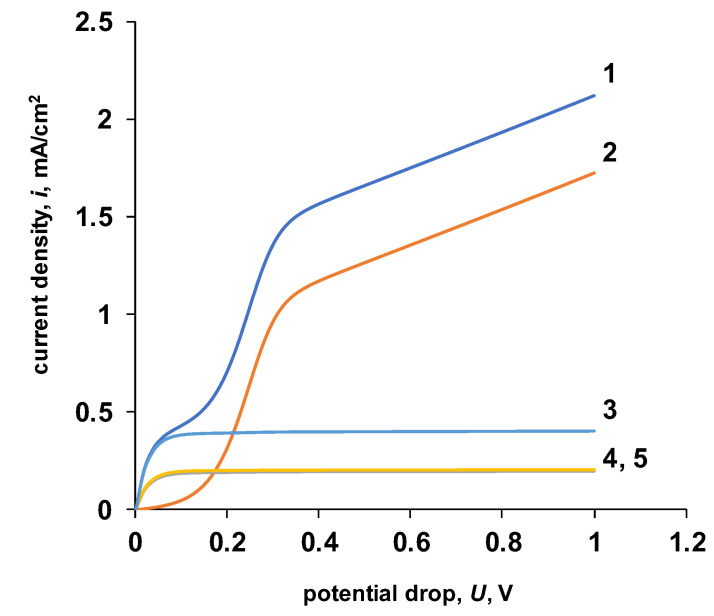
The initial section of the calculated general current–voltage characteristic. 1—general current–voltage characteristic, 2—total current for water dissociation products, 3—total current for salt ions, 4—current for salt anions, 5—current for salt cations. The values of the parameters given in Table 2 are used for the calculation. $k_{2}^{*}$ = 10 M/s, *β* = 4 m/GV.

Let us consider in more detail the initial section of the current–voltage characteristic (Figure 10). An analysis of partial currents for salt ions and water dissociation products shows that in the initial section of the current–voltage characteristic, there is a sharp decrease in the current growth rate for water dissociation products. Let us consider the possible causes of these effects.

**Figure 11:**
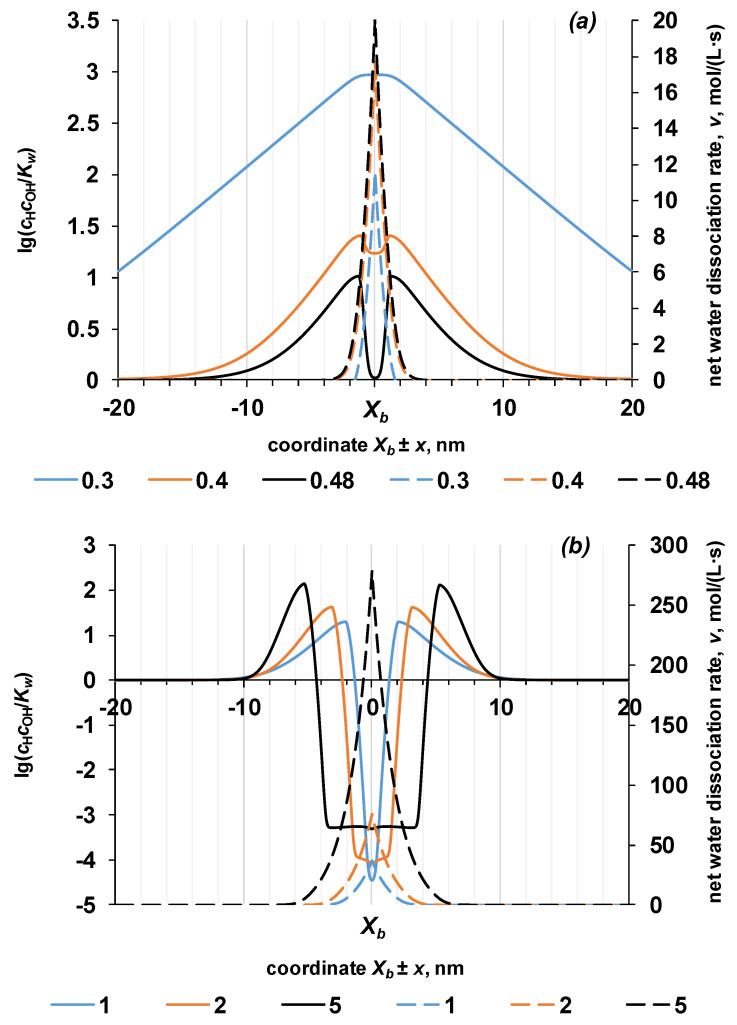
The normalized product of the concentrations of hydrogen and hydroxyl ions (solid lines) and the net rate of the water dissociation reaction (dashed lines) near the point $x=X_{b}$ at small (**a**) and large (**b**) potential drops (numbers in the legend). The values of the parameters given in Table 2 are used for the calculation. $k_{2}^{*}$ = 10 M/s, *β* = 4 m/GV.

The reason for this dependence may be related to the change in the rate of the water dissociation reaction as the space charge region expands. At a relatively small potential drops, the space charge region is rather narrow, and a large number of charge carriers appear near the *X_b_* boundary as a result of the water dissociation reaction. The resulting hydrogen and hydroxyl ions are removed from the reaction zone under the action of gradients of the electric and concentration fields (Figure 11a). Thus, the occurrence of the reaction of water dissociation at the bipolar boundary with the participation of ionogenic groups of the membrane violates the condition of local chemical equilibrium (*K_w_* = *c*_H^+^_*c*_OH^+^_ = 10^−14^) and the value of *K_w_* can be almost three orders of magnitude higher than the equilibrium value (under the conditions of the particular numerical experiment).

A further increase of the potential drop leads to an expansion of the space charge region, and with it, the thickness of the zone in which the water dissociation reaction proceeds. One can observed that in the region closest to the bipolar boundary, the product of the concentrations of hydrogen and hydroxyl ions decrease from the maximum values. With a potential drop of 0.48 V, a decrease in the concentration product is observed in the region $x=X_{b}\pm 1\;\mathrm{nm}$ to a value comparable to the equilibrium value of the ionic product of water. A further increase in the potential drop leads to a decrease in the ion product below the equilibrium values. At the same time, the net rate of the water dissociation reaction only increases as the potential drop increases.

The calculated rate profile of the water dissociation reaction depends almost linearly on the coordinate when considering the process in the direction from the bipolar boundary into the bulk of the monopolar ion-exchange layer. The maximum value of the reaction rate is localized at the point with the maximum electric field strength. Thus, a larger number of hydrogen and hydroxyl ions are formed at the bipolar boundary; however, even at some distance away from the bipolar boundary, the reaction proceeds at a non-zero rate. For example, in the particular case under consideration with a potential drop of 5 V, the non-zero value of the reaction rate is retained in a layer 12 nm thick, 6 nm in each monopolar layer. Due to the water dissociation reaction, the formed protons move to the left and the hydroxyl ions move to the right (Figure 3). This condition is true for each point at which the reaction proceeds. Thus, the flow of ions formed at the bipolar boundary moves through the reaction zone through a counter flow of ions with which they possess high chemical affinity. For example, hydrogen ions move through a stream of hydroxyl ions and vice versa. Since the concentration of hydrogen and hydroxyl ions becomes much higher than the equilibrium value (10^−7^ M), most of these ions recombine into water molecules. Only those ions remain, which appear because of the dissociation reaction at the outer boundaries of the space-charge region and which are quickly removed outside the reaction zone. This is indirectly evidenced by the fact that the width of the region in which the local quasi-equilibrium is disturbed is much larger than the reaction region (Figure 11b). 

The electric field strength decreases with distance from the interface, i.e., the reaction rate of water dissociation, at the boundary of the reaction zone, is lower than directly at the interface. Based on the foregoing, it can be argued that the second inflection on the calculated total current–voltage curve (Figure 10) appears because of a decrease in the flux of H^+^/OH^−^ ions, which is caused by partial recombination of charge carriers inside the reaction zone. This inflection on the current–voltage curve is the “apparent” limiting current for the products of water dissociation.

Separately, it should be noted that, to date, the current–voltage characteristics of bipolar, bilayer, or multilayer membranes have not been experimentally obtained that correspond in shape to the calculated current–voltage characteristic. A somewhat similar current–voltage characteristic, also containing two inflections, was obtained in [16]. However, the authors of this work explained the second inflection by the phenomenon of current induced membrane discharge, that is, an increase in the flux of salt ions through the bipolar membrane because of a decrease in its ion-exchange capacity. It should be noted that the loss of selectivity for salt ions (i.e., an increase in their flux) in acidic media is indeed possible for the MB-3 membrane, and it has been repeatedly observed experimentally [24,63], however, the reported current–voltage characteristics of these membranes [64,65] do not possess such features.

### 4.1. Influence of Model Parameters on the Shape and Characteristic Points of the Current–Voltage Characteristic

The “apparent” limiting current by the products of water dissociation reaction should depend on the rate of the water dissociation reaction; its value should depend on the kinetic parameters of this reaction, namely, on the parameters *k*_2_ and *β*.

**Figure 12:**
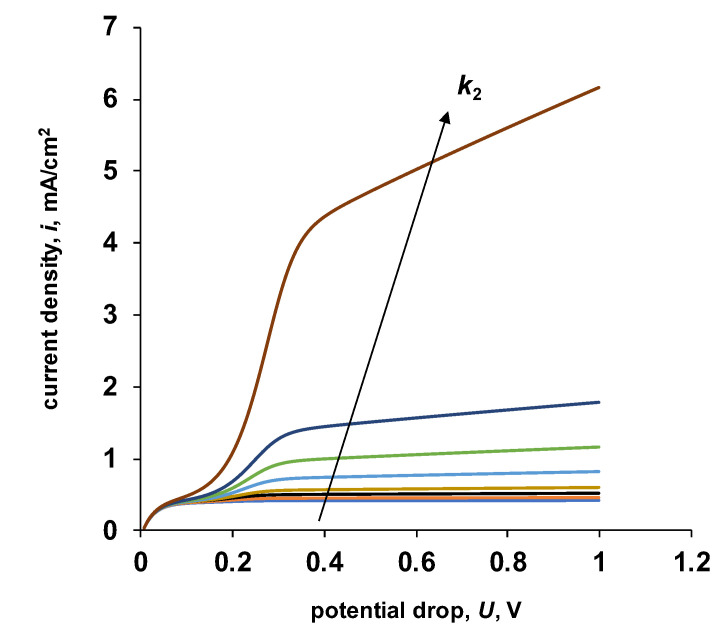
The initial sections of the calculated current–voltage characteristics of a bipolar membrane at various values of the parameter k2. For the calculation, the values of the parameters given in Table 2 were used. *β* = 1 m/GV, *k*_2_ = 0.2, 0.5, 1, 2, 5, 10, 20, 100 M/s.

On the calculated current–voltage characteristics at different values of the parameter *k*_2_, an increase in the value of the “apparent” limiting current with an increase in the parameter is observed (Figure 12). The limiting current for salt ions remains constant for all numerical experiments (Table 4).

**Figure 13:**
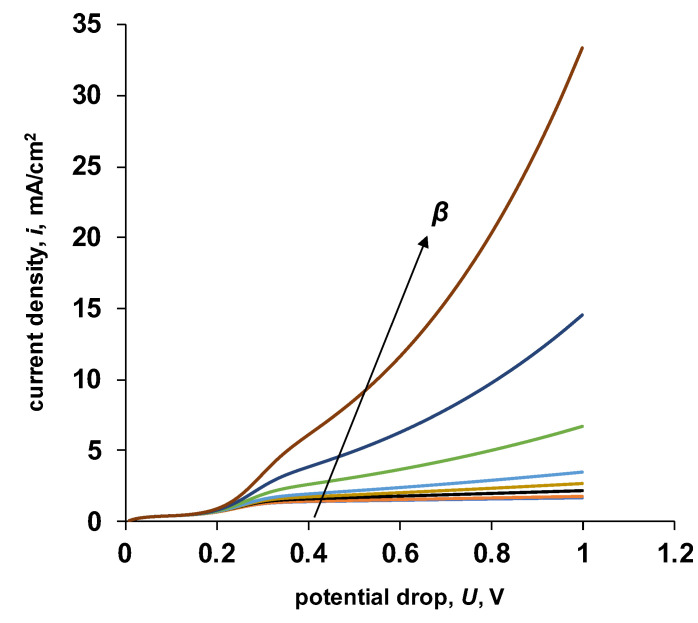
Initial sections of the calculated current–voltage characteristics of a bipolar membrane for various values of the entropy factor. For the calculation, the values of the parameters given in Table 1 were used. *k*_2_ = 20 M/s, *β* = 0.5, 1, 2, 3, 4, 6, 8, 10 m/GV.

Despite the change in the parameter *k*_2_, the increase in current on the general current–voltage curve after reaching the “apparent” limiting current is linear. Based on the accepted exponential nature of the dependence of the water dissociation reaction rate (Equation (21)), the entropy factor (parameter *β*) influences the steepness of the overlimiting section of the current–voltage characteristic. The results of calculating the current–voltage characteristics for different values of the parameter *β* are shown in Figure 13.

As can be seen from the results obtained, the parameter *β* does not only affect the steepness of the overlimiting section. With an increase in the value of the parameter *β*, the slope of the section of the current–voltage characteristic, located between the limiting current for salt ions and the “apparent” limiting current for water dissociation products, increases (Table 5).

Mareev et al. in [16] proposed to relate the entropy factor *β* to the Bjerrum length (*l_b_*) by the following simple relation (the Bjerrum length is the distance between two charges at which the force of electrostatic interaction balances the force of thermal motion of ions):

(31) $$\beta =\frac{F}{RT}l_{b}$$

Using the data on the value of the parameter *β* for various bipolar membranes, let us try to estimate the value of the Bjerrum length for them using Equation (31). The values of the parameter *β* and the calculation results are shown in Table 6.

As can be seen from the data in the table, the value of *l_b_* calculated for the known values of the parameter *β* for bipolar membranes is several times smaller than the Bjerrum length found for an aqueous solution. Mafe et al. [32] suggested the use of a characteristic length *α* (also suggested by Timashev and Kirganova [28]). Mafe et al. assumed that this length parameter is the distance at which the water dipole must approach the ionogenic group in order for the potential barrier of proton transfer from the ionogenic group to the water molecule to become lower than in pure water. Mafe et al. predicted the value of *α* to be 0.27 nm.

It seems more general to consider the parameter *β* as a certain characteristic length. The larger the parameter *β*, the higher the probability that the counterion (hydrogen or hydroxil ion) formed because of the water dissociation reaction is located near the ionogenic group. Kamcev et al. [66,67] proposed to use Manning’s condensation theory for ion-exchange membranes. According to [66,67], counterions that are in a “condensed” state near the ionogenic group are more mobile (because they are in a state with a minimum potential energy) and can easily be transferred along the ion-polymer chains of an ion exchanger in the so-called low-potential tunnel. It is possible that the products of water dissociation located inside the potential tunnel are quickly removed from the reaction zone under the action of an electric field and do not participate in recombination reactions. At the same time, ions outside the potential tunnel are more likely to recombine back into a water molecule. If the value of *β* is sufficiently large (comparable to the width of the ion channel), then a situation is possible when all ions at the cation-exchanger/anion-exchanger interface are inside the potential tunnel (the case when the characteristic length is equal to the diameter of the ion channel), then the ion recombination reactions occur quite rarely, which is expressed in the growth of the flow of products of the water dissociation reaction. This interpretation of the parameter *β* makes it possible to relate it to the molecular structure of the bipolar boundary.

### 4.2. Comparison of Numerical Simulation with Experimental Data

**Figure 14:**
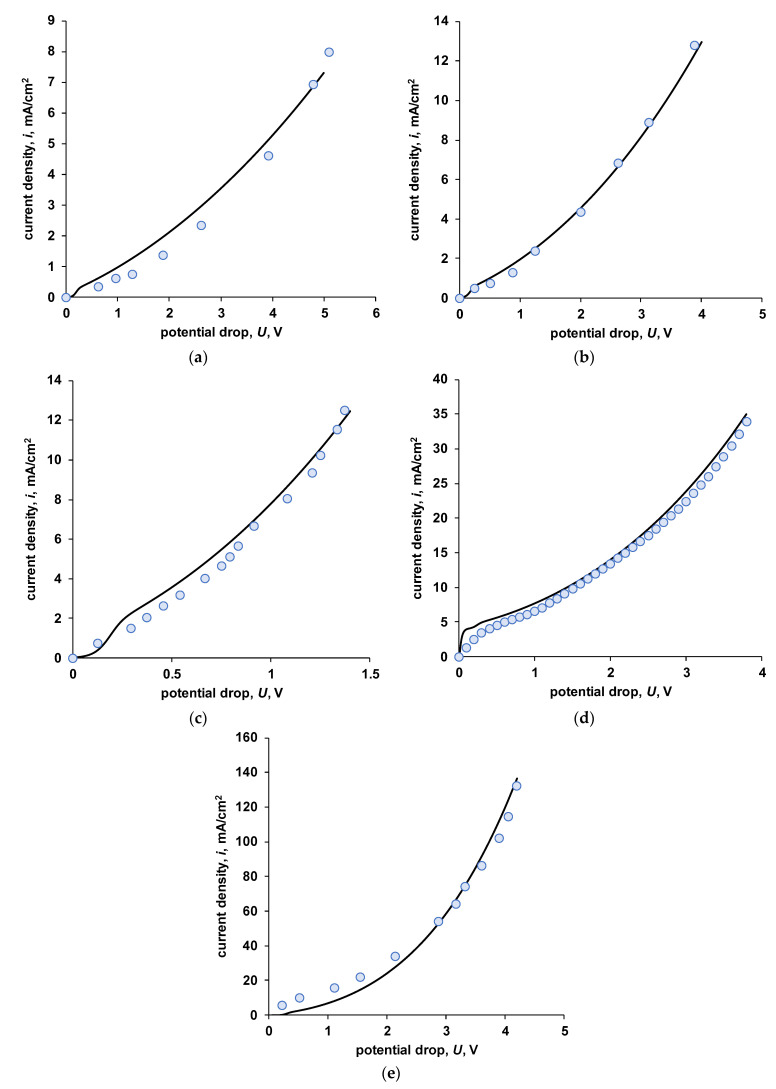
Calculated (lines) and measured (dots) total current–voltage curves for various electromembrane systems with bipolar membranes. (**a**)—MB-2 in 0.01 M NaCl, (**b**)—MB-1 in 0.01 M NaCl, (**c**)—MB-3 in 0.01 M NaCl, (**d**)—MB-3 in 0.5 M NaCl, (**e**)—MB-3 in 0.1 M NaCl (data adapted from [44]).

Experimental current–voltage characteristics of various heterogeneous bipolar membranes are presented in Figure 14. The results of numerical simulation are also shown there.

The MB-2 membrane is a bipolar membrane in which the cation-exchange and anion-exchange layers do not contain catalytically active ionogenic groups. In the absence of catalytic additives introduced into the bipolar region (for example, as in [24,45]), this membrane has a high operating voltage, which is clearly seen from Figure 14a. Table 7 shows the model parameters that provide a fairly good agreement between the experimental and calculated current–voltage characteristics. Since the MB-2 membrane contains strongly acidic and strongly basic ionogenic groups, and also considering the low concentration of the salt solution, it is not possible to establish the value of the limiting current for salt ions on the experimental curve. The same dependence is observed for the numerical calculation. However, in the latter case, it is possible to fix a certain value of the pseudolimiting current from the products of the water dissociation reaction.

The MB-1 membrane (Figure 14b) contains tertiary and secondary amino groups in the anion-exchange layer which determines its moderate activity in the water dissociation reaction. Since weakly basic ionogenic groups have a lower selectivity (the ability to retain salt ions), compared to strongly basic ones, one can see the limiting current for salt ions on the general current–voltage characteristics of this membrane. This membrane has a significant asymmetry of properties: the catalytic activity of ionogenic groups in the cation-exchange and anion-exchange layers and the exchange capacity differ. In this regard, direct application of the developed model is somewhat limited.

The MB-3 membrane is similar in many respects to the MB-1 membrane, with the difference that the ionogenic groups in the cation-exchange layer are more active in the reaction of water dissociation. The calculation results and experimental data obtained for salt solutions of various concentrations are presented in Figure 14c–e. It can be seen that with the correct selection of the model parameters, it is possible to achieve a good agreement between the experimental and calculated curves.

Separately, the case when the MB-3 membrane is in a 0.5 M sodium chloride solution is knocked out. In this case, the calculation using parameters close to other cases ($c_{f}^{c}$ = 3 M, $c_{f}^{a}$ = 1 M) leads to a significantly overestimated value of the limiting current for salt ions. In addition, the rate constant of the rate-limiting step of the water dissociation reaction involving the ionogenic groups of the membrane and the value of the entropy factor are lower than in other cases. The experimental results can be explained by the high diffusion permeability of the cation-exchange layer of the MB-3 membrane for salt ions. As a result, the dissociation reaction in concentrated solutions starts at significantly higher potentials. As a result, even for an approximate description of the current–voltage characteristic (Figure 14d), it is required to use unrealistic values of the model parameters.

In general, when comparing the results of numerical calculations with experiment, it can be concluded that the selected parameter values are within reasonable assumptions (except for the case of the MB-3 membrane in 0.5 M NaCl). The values of the $k_{2}^{*}$ rate constants increase in the series MB-2 < MB-1 < MB-3, which is in good agreement with the known experimental and literature data. The values of the entropy factor (β) also lie in the range known from the literature ≈ 3–6 m/GV.

## 5. Conclusions

A model is proposed that describes the transfer of ions and the process of water dissociation in a system with a bipolar membrane and adjacent diffusion layers. The model considers the transfer of four types of ions: the cation and anion of salt and the products of water dissociation—hydrogen and hydroxyl ions. To describe the process of water dissociation, a model for accelerating the dissociation reaction with the participation of ionogenic groups of the membrane (the model of catalysis of the reaction by ionogenic groups) is adopted. The COMSOL Multiphysics 5.5 software package was used to solve the boundary value problem. For a symmetric electromembrane system, a solution to the boundary problem was obtained: the dependences of the fluxes of all ions in the electromembrane system, the concentration profiles of ions in each of the layers, the potential distribution profiles, the electric field strength, and the dissociation and recombination reaction rates of water molecules.

An analysis of the results of a numerical experiment allows us to show that, at least in a symmetric electromembrane system, there is a kinetic limitation of the water dissociation process, apparently associated with the occurrence of recombination reactions of dissociation products in the region of the bipolar boundary. The existence of such kinetic limitations is expressed in the appearance of a second bend in the calculated total current–voltage characteristic of the membrane, which can be called the “apparent” limiting current in terms of water dissociation products. The magnitude of the “apparent” limiting current depends on the kinetic characteristics of the process of water dissociation—the rate constant of the limiting stage of the reaction involving the ionogenic groups of the membrane and the entropy factor.

The entropy factor—the parameter *β*—can be interpreted as a characteristic length, which shows the possibility of an ion that appeared because of the water dissociation reaction to be removed from the reaction zone without participating in recombination reactions. To verify this hypothesis, experimental studies of bipolar membranes with a well-known (or, in the ideal case, specified at the synthesis stage) structure of ion-polymer chains in the space charge region are required.

In the future, it is planned to refine the methods for solving the boundary value problem in order to take into account a number of effects that accompany the process of water dissociation in bipolar membranes: charge asymmetry of the cation-exchange and anion-exchange layers; asymmetry of the thickness of monopolar layers; asymmetry in the composition of solutions on both sides of the membrane (transition from the salt system to the “acid-base” system, which is closer to the real aspects of the functioning of bipolar membranes); taking into account changes in the dielectric constant of the medium in the region of the water dissociation reaction; taking into account the protonation/deprotonation of the ionogenic groups of the membrane and the influence of a high field strength on this process; diffusion of water molecules to the bipolar boundary. Considering even some of the above assumptions will significantly improve the understanding of the processes of ion transfer and the reaction of water dissociation in electromembrane systems with bipolar membranes.

## Figures and Tables

**Table 1:** Catalytic activity of various fixed groups in the water dissociation reaction.

Fixed Group	−SO_3_H	>	−COOH	>	−PO(OH)_2_
p*K*_a_	1 ÷ 2		4 ÷ 6		3.5; 7
Δ*φ* (4 A/dm^2^), V	4 ÷ 18		2.0 ÷ 3.2		1.1 ÷ 1.7

**Table 2:** Initial parameters of the symmetrical model.

Model Parameter	Symbol	Value
Thickness, μm	*d_a_*	450
	*d_c_*	450
	*δ*	100
Concentration, M	*c_s_*	0.1
	$c_{H}$, $c_{\mathrm{OH}}$	10^−7^
	$c_{f}^{c}$, $c_{f}^{a}$	1
Diffusion coefficients in solution, cm^2^/s [57]	$D_{C}$	1.9 × 10^−5^
	$D_{A}$	2.032 × 10^−5^
	$D_{H}$	9.312 × 10^−5^
	$D_{\mathrm{OH}}$	5.26 × 10^−5^
The diffusion coefficients in the membrane are taken to be an order of magnitude smaller than in solution		
Water recombination rate constant, M^−1^·s^−1^ [58]	$k_{r}$	1.3 × 10^11^
Water dissociation rate constant, s^–1^ [58]	$k_{d}$	2.5 × 10^−5^
Ionic product of water, M^2^ [58]	$K_{w}$	1 × 10^14^

**Table 3:** Physicochemical properties of studied bipolar membranes.

Property	Membrane		
	MB-1	MB-2	MB-3
Polymeric matrix	Polystyrene divinylbenzene (cation-excahnge layer) Epichlorohydrin with polyethelenepolyamines (anion-excahnge layer)	Polystyrene divinylbenzene	
Inert binder	Polyethylene		
Reinforcing mesh	Polyamide		
Wet thickness *, mm	0.9 ± 0.3		
Ion-exchange capacity *, mmol/g-dry:			
cation-exchange layer	1.4	1.4	1.9
anion-exchange layer	4.3	1.4	1.4
Ionic group:			
cation-exchange layer	−SO_3_H	−SO_3_H	−PO_3_H_2_
anion-exchange layer	≡N, =NH	−N(CH_3_)_3_	−N(CH_3_)_3_

* ion-exchange capacities of bipolar membranes are taken from [30].

**Table 4:** The values of the limiting currents found from the calculated current–voltage curves for different values of the parameter $k_{2}^{*}$.

Limiting Current	Effective Water Dissociation Constant, $k_{2}^{*}$, M/s							
	0.2	0.5	1	2	5	10	20	100
$i_{\lim}^{s}$, mA/cm^2^	0.39	0.39	0.39	0.39	0.39	0.39	0.39	0.39
$i_{\lim}^{W}$, mA/cm^2^	0.43	0.45	0.5	0.58	0.74	1.0	1.4	4.3

**Table 5:** The values of the limiting currents found from the calculated current–voltage curves for different values of the parameter *β*.

Limiting Current	Entropy Factor, β, m/GV							
	0.5	1	2	3	4	6	8	10
$i_{\lim}^{s}$, mA/cm^2^	0.39	0.39	0.39	0.39	0.39	0.39	0.39	0.39
$i_{\lim}^{W}$, mA/cm^2^	1.37	1.40	1.90	1.70	1.84	2.30	2.90	4.80

**Table 6:** The values of the parameter *β* and the value of the Bjerrum (characteristic) length calculated by Equation (31) for bipolar membranes.

Property	Membrane							
	MB-1 [30]	MB-2 [30]	MB-3 [30]	MB-2m [24]	BM-a-30 [31]	MB-2_Cr [45]	BM-1 * [19]	Water
Water dissociation activity	medium	low	high	high	low	high	high	-
β, m/GV	3.65	7.17	6.41	5.2	5.16	8.62	13.4	-
*l_b_* (*α*), nm	0.10	0.18	0.16	0.13	0.13	0.22	0.34	0.7

* the value of the parameter was calculated based on the experimental data given in the paper [19].

**Table 7:** Values of the model parameters used for fitting of the experimental data.

Parameter/Variable	Membrane				
	MB-2 (Figure 14a)	MB-1 (Figure 14b)	MB-3 (Figure 14c)	MB-3 (Figure 14d)	MB-3 (Figure 14e) [44]
*d_c_*, μm	450	450	450	450	450
*d_a_*, μm	450	450	450	450	450
*δ*, μm	53	53	53	53	unknown
*c_s_* (NaCl), M	0.01	0.01	0.01	0.5	0.1
$c_{f}^{c}$, M	1	1	3	5.5	3
$c_{f}^{a}$, M	1	2	1	5.5	1
water dissociation layer *	CEL and AEL	AEL	CEL	CEL	CEL
$k_{2}$, s^−1^	0.4	0.7	1.2	0.5	1.8
$k_{2}^{*}$, M/s	20	80	198	165	300
β, m/GV	2.8	3.4	5	2.2	6.4

* indicates by which layer of the bipolar membrane the water dissociation reaction is catalyzed.

## Data Availability

The data presented in this study are available on request from the corresponding author.

## Abbreviations

**Symbol**	**Dimension Description**
d_a_, d_c_, δ	m Thickness of anion-exchange layer, cation-exchange layer and diffusion layer, respectively
$j_{i}^{k}$	mol/(m^2^∙s) The flux of ion i in the layer k
$D_{i}$	m^2^/s The diffusion coefficient of ion i
$c_{i}$	mol/m^3^ The concentration of ion i
φ	V The electric potential
x	m The coordinate
$\nu^{cat}$	mol/(m^3^∙s) The net water dissociation reaction rate
$c_{f}^{j}$	mol/m^3^ The concentration of fixed ions in the membrane layer j (j = c for cation-exchange layer and j = a for anion exchange layer)
$\varepsilon_{0}$	F/m The absolute permittivity
ε	The relative permittivity
$K_{w}$	mol^2^/m^6^ The ionic product of water
U	V The potential drop
i	A/m^2^ The current density
$z_{i}$	The charge of ion i
$X_{l},X_{r},X_{b}$	The boundaries cation-exchange layer/solution, anion-exchange layer/solution, cation-exchange layer/anion-exchange layer, respectively
$k_{2},k_{-2}$	m^6^/(mol^2^∙s) The forward and backward rate constants of the water dissociation limiting stage involving ionic groups of the membrane
$k_{d},k_{r}$	m^6^/(mol^2^∙s) The forward (dissociation) and backward (recombination) rate constants of water autoprotolysis
*E*	V/m The electric field strength
β	m/V The entropy factor
$c_{s}$	mol/m^3^ The concentration of salt ions
$c_{W}$	mol/m^3^ The concentration of protons and hydroxyl ions
$\lambda_{\mathrm{CEL}}+\lambda_{\mathrm{AEL}}$	m The thickness of the space charge region
$i_{\lim}^{s}$	A/m^2^ The limiting current by salt ions
$i_{\lim}^{W}$	A/m^2^ The apparent limiting current by water dissociation
$l_{b}$	m The Bjerrum length
F	C/mol The Faraday constant
R	J/(mol∙K) The universal gas constant
T	K The absolute temperature
